# Locally advanced anorectal malignant melanoma in septuagenarian patient treated by laparoscopic abdominoperineal resection: A case report

**DOI:** 10.1016/j.ijscr.2021.106378

**Published:** 2021-09-06

**Authors:** Takuya Futori, Tsuyoshi Enomoto, Yohei Owada, Yusuke Ohara, Hideki Matsumura, Tatsuya Oda

**Affiliations:** University of Tsukuba, Faculty of Medicine, Department of Gastrointestinal and Hepato-Biliary-Pancreatic Surgery, 1-1-1 Tennnodai, Tsukuba-shi, Ibaraki-ken 305-8575, Japan

**Keywords:** Anorectal malignant melanoma, Laparoscopic abdominoperineal resection, Septuagenarian, Case report

## Abstract

**Introduction:**

Anorectal malignant melanoma (ARMM) is a rare disease with a poor prognosis. In cases involving locally advanced disease, the treatment strategy is difficult, especially in octogenarian patients, because the prognosis is poor, despite the corresponding decrease or loss of the anal function.

**Presentation of case:**

A 78-year-old woman was admitted to a local hospital with chief complaints of severe anal discomfort due to an egg-sized tumor that was protruding out of the anus and melena. A diagnosis of ARMM was confirmed based on the examination of biopsy specimens and imaging study showed swollen lymph nodes on the dorsal side of the middle rectum and left internal iliac lymph nodes.

Laparoscopic abdominoperineal resection with left lateral lymph node dissection was performed. The examination of the resected specimen revealed two polypoid tumors with a maximum diameter of 38 mm and 14 mm with a metastatic lymph node of 62 mm in the mesorectum. The postoperative course was uneventful. Relapse and local recurrence free survival without any complaints was obtained for more than 12 months.

**Discussion:**

With respect to locoregional disease control, it has been reported that abdominoperineal resection can obtain better control of local disease in comparison to local resection. Laparoscopic surgery is advantageous in its facilitation of an early postoperative recovery for elderly patient.

**Conclusion:**

Laparoscopic abdominoperineal resection may control locoregional disease and improve the patient's QOL with early postoperative recovery. —even in septuagenarian patients—may become a treatment strategy for advanced ARMM.

## Highlights

• Anorectal malignant melanoma is a rare disease with a poor prognosis.
• Surgical procedure for anorectal malignant melanoma is controversial.
• APR may achieve better local control for advanced anorectal malignant melanoma.
• Laparoscopic surgery is advantageous in its facilitation of an early recovery for elderly patient.

## 1 Introduction and importance

Anorectal malignant melanoma (ARMM) is a rare disease derived from the squamous zone and the anal transitional epithelium of the anoderma, with the inclusion of melanocytes; it accounts for approximately 0.5–1.6% of all anorectal malignancies [1]. ARMM is characterized by aggressive behavior with high metastatic potential in the early postoperative periods and a poor prognosis, with a 5-year survival rate of 6–28.8% [2]. Regardless of the aggressive nature of the disease and its poor prognosis, in cases involving advanced-stage ARMM, abdominoperineal resection (APR) is required as treatment for long term survive as well as for the major control of clinical symptoms from locoregional disease [3]. Thus, considering the poor prognosis and reduced QOL after abdominoperineal resection, decision-making regarding the appropriate treatment strategy is extremely difficult for elderly patients, especially for octogenarians.

We herein report a case of advanced ARMM in an octogenarian patient who was successfully treated with laparoscopic APR with pelvic lateral lymph node dissection, which controlled the patient's locoregional complaints and achieved a relapse-free status. We also review the relevant literature. This report is in line with the SCARE criteria [4].

## 2 Case presentation

**Fig. 1:**
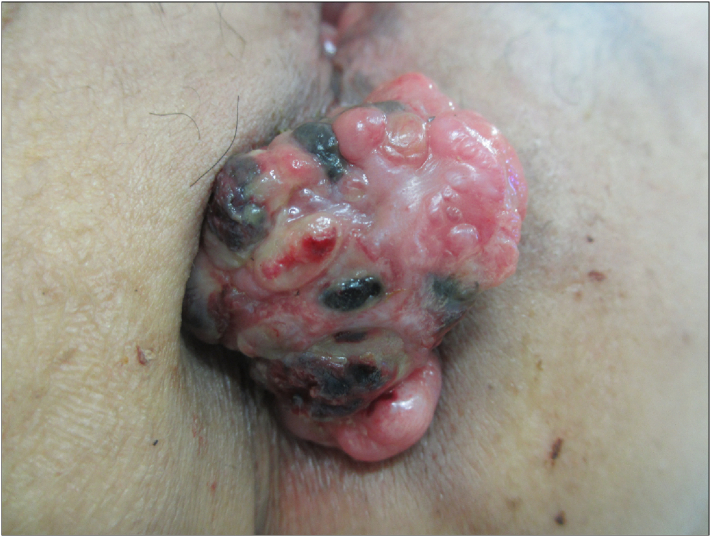
An egg-sized mass of approximately 30 mm in diameter with an irregular surface protruded from the anal canal to outside the anus.

A 78-year-old female was admitted neighbor hospital with chief complaints of anal discomfort and melena. Her history of illness included diabetes mellitus and hypertension, which were medically treated. She was otherwise healthy. A physical examination revealed an egg size tumor of approximately 30 mm in diameter protruding from the anal canal, past the anus (Fig. 1). A diagnosis of malignant melanoma was confirmed based on the examination of biopsy specimens, and she was introduced to our department for further treatment.

**Fig. 2:**
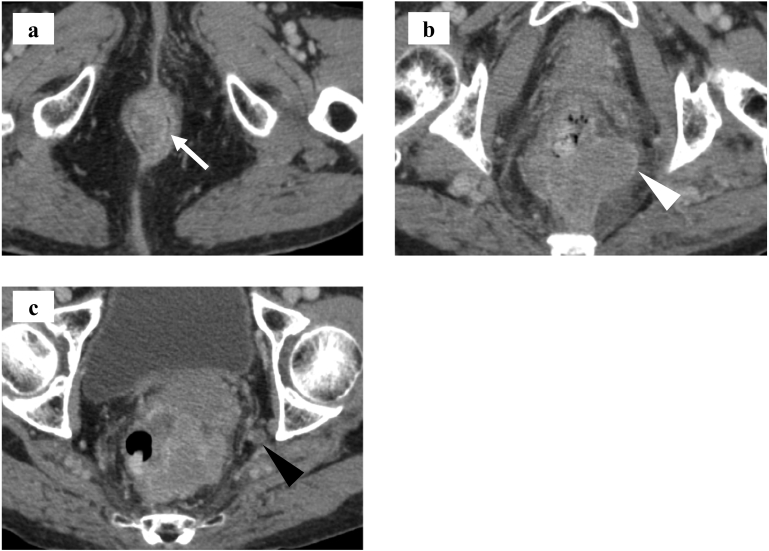
(a) Enhanced computed tomography demonstrated two polypoid tumors with a maximum diameter of 38 in the lower rectum (white arrowhead). (b) A metastatic lymph node of 50 mm in diameter was found in the mesorectum of the upper rectum. (c) A lymph node of 8 mm in diameter in the left internal iliac region (black arrowhead).

A laboratory analysis revealed an elevated serum CEA value (9.3 ng/ml); her other values were within the normal range. Colonoscopy revealed an egg-sized tumor of 30 mm in diameter protruding from the anal verge, and small elevated lesions with some black pigmentation in the rectal mucosa next to the anal ring. Contrast-enhanced computed tomography and MRI revealed a lobular-shaped lymph node of 50 mm in diameter in the mesorectum near the tumor and a lymph node of 8 mm in diameter in the left internal iliac region **(**Fig. 2a, b, c**)**. Based on the above findings, a preoperative diagnosis of anorectal malignant melanoma with regional lymph nodes metastasis, cT1N1bM0, cStage IIIA according to TNM staging of rectal cancer based on the 7th AJCC staging system, was made. Treatment strategy was discussed and determined by multidisciplinary team assessment including by surgeon, gastroenterologists, radiation oncologists and dermatologists.

**Fig. 3:**
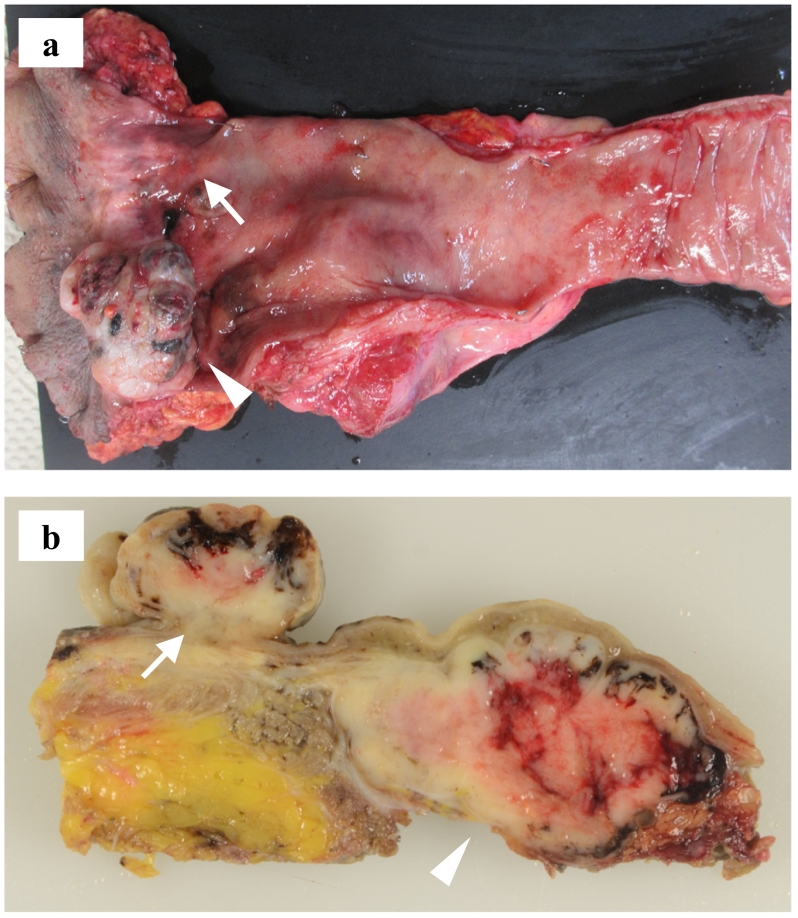
(a) The examination of the resected specimen revealed a polypoid shaped tumor of 38 mm (white arrowhead) and a 14-mm elevated lesion type tumor (white arrow) near the dentate line, lymph node metastasis of 50 mm in size was also observed in the mesorectum of the upper rectum. (b) Examination of the cut section showed that the inside of the tumor included a yellow solid component with partially blackish brown components. (For interpretation of the references to colour in this figure legend, the reader is referred to the web version of this article.)

**Fig. 4:**
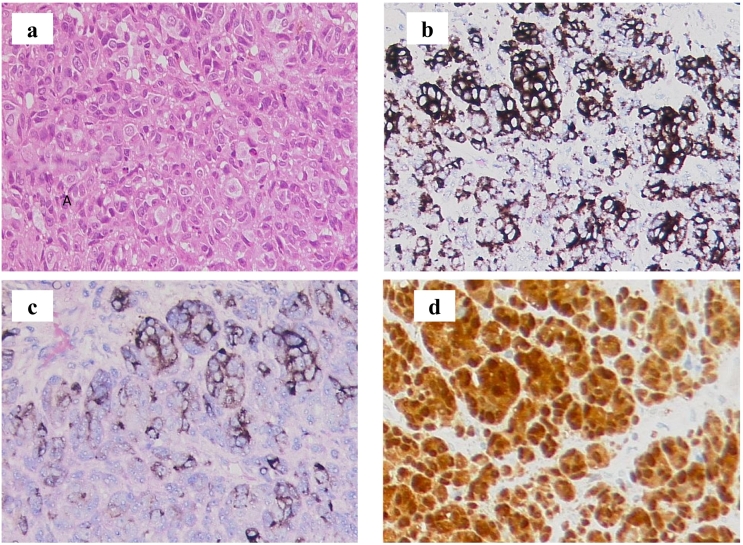
(a) A histopathological examination showed spindle-shaped, small ellipsoidal heterozygous proliferation with melanin. (b) Immunohistological staining was positive for HMB 45 (x200), (c) melan-a (x200), (d) S100 (x200) and Vimentin, and negative for Pan-Keratin and CD45.

Laparoscopic APR with autonomic nerve preserving left lateral lymph node dissection was performed. The operative time was 365 min, and the amount of bleeding was 10 ml. The examination of the resected specimen revealed a polypoid-shaped tumor of 38 mm in diameter and an elevated lesion-type tumor of 14 mm near the dentate line; a metastatic lymph node of 50 mm in size was also observed in the mesorectum of the upper rectum. The examination of the cut section showed that the inside of the tumor included a yellow solid component with partially blackish brown components **(**Fig. 3a, b**)**. A histopathological examination revealed spindle-shaped, small ellipsoidal heterozygous proliferation with melanin, and other immunohistological staining revealed positivity for HMB 45, melan-a, S100 and Vimentin, negativity for Pan-Keratin and CD45 **(**Figure4a, 4b, 4c, 4d**).** Based on the above findings, the diagnosis was malignant melanoma of the rectum, pT2N1aM0, pStage IIIA.

The postoperative course was uneventful, and the patient was discharged on the 25th postoperative day without any adverse events. The patient's quality of life was maintained without any anal discomfort or locoregional relapse by clinical and image findings for 24 months after the operation.

## 3 Clinical discussion

Determining the appropriate surgical treatment for advanced ARMN in elderly patients with severe symptoms is difficult because its poor prognosis despite of the decrease or loss of the anal and genito-uretheral function after surgery. When a patient with ARMM undergoes surgical treatment, especially APR, an early postoperative of without any adverse events and a quick return to normal life is desirable.

The reported five-year survival rate after radical resection of ARMM is 6–28.8% [2]. A study from the Netherlands showed that the median survival time varies according to stage; the median survival time of patients with stage I, II, and III disease was 28 months, 16 months, and 4 months, respectively [5]. However, all reports were based on retrospective studies and only included limited numbers of patients because of the rarity of this disease.

One of the reasons for the poor prognosis of ARMM may be attributed to the difficulty in its diagnosis. In the early disease stage, a lack of symptoms and the characteristic pigmented lesions are usually located in hidden areas. Furthermore, ARMN have the aggressiveness of the tumor biology and closely contact with the rich lympho-vascular supply of the underlying anorectal mucosa, it would contribute to the high potential to metastasize to the liver, lungs, skin, and brain, as well as the mesenteric, inguinal, and hypogastric paraaortic lymph nodes [6], [7], [8]. Thus, the majority of ARMM patients have advanced lesions of >2 mm in thickness and the incidence of locoregional lymph node metastasis is approximately 61%, while distant metastasis is identified in as many as 29% of patients on initial presentation [9].

APR and wide local excision (WLE) are performed for the surgical treatment of ARMM. The appropriate surgical procedure remains controversial because the overall survival and recurrence-free survival of patients treated with these procedures do not differ to a statistically significant extent [10]. However, when planning surgical treatment, it may be important to improve the patient's symptoms and maximizing quality of life after operation. With respect to locoregional disease control, it has been reported that APR can obtain larger negative margins and achieve better control of local disease in comparison to WLE [11], [12], [13]. Thus, APR would seem to be an appropriate treatment for ARMM with a tumor thickness of >4 mm. In contrast, for early lesions with a tumor thickness of ≤4 mm, a wide local excision with a sphincter preserving local excision with a 1 to 2-cm margin is recommended [14].

Recently, ARMM has been treated by laparoscopic APR [11], [15]. Laparoscopic rectal surgery is considered to be less invasive, and is associated with various advantages, including early postoperative recovery of the bowel function, a reduce time to the resumption of a normal diet and time to first defecation, as well as an improved physical function with less fatigue and fewer pelvic as urine function at three months after operation [16], [17]. Thus, a laparoscopic approach can be safely performed whatever the age of patient, therefore it may be useful than open surgery for elderly patients [18].

## 4 Conclusion

In our case, the ARMM was locally large, and was infiltrating into a deeper part of the submucosal layer, with a tumor thickness of >30 mm with positive lymph node metastasis, with protrusion from the anus. Surgical treatment would be considered about a low risk of local failure and a life free of severe discomfort. Laparoscopic APR may be reduced postoperative morbidity and enable better locoregional control to be achieved with an improvement in the quality of life, even in octogenarian patients. Laparoscopic APR, which may control locoregional disease and improve the patient's QOL—even in octogenarian patients—may become a treatment strategy for advanced ARMM. T.

## Consent

Written informed consent was obtained from the patient and her family for the publication of this case report and the accompanying images. A copy of the written consent is available for review by the Editor in Chief of this journal upon request.

## Sources of funding

None.

## Ethical approval

This report is not a research study.

## Guarantor

Tsuyoshi ENOMOTO.

## Provenance and peer review

Not commissioned, externally peer-reviewed.

## CRediT authorship contribution statement

TF contributed to play important role in clinical setting and wrote of the manuscript. TE is the corresponding author and conceptualization of treatment and the manuscript. All authors revised and approved the final manuscript.

## Declaration of competing interest

All authors declare that they have no conflict of interests.
